# A cost and community perspective on the barriers to microbiome data reuse

**DOI:** 10.3389/fbinf.2025.1585717

**Published:** 2025-04-09

**Authors:** Julia M. Kelliher, Leah Y. D. Johnson, Francisca E. Rodriguez, Jaclyn K. Saunders, Marie E. Kroeger, Buck Hanson, Aaron J. Robinson, Winston E. Anthony, Marc W. Van Goethem, Anders Kiledal, Ahmed A. Shibl, Amanda Araujo Serrao de Andrade, Cassandra L. Ettinger, Chhedi Lal Gupta, Chris R. P. Robinson, Cristal Zuniga, Daniel Sprockett, Douglas Terra Machado, Emilie J. Skoog, Iyanu Oduwole, Jason A. Rothman, Kaelan Prime, Katherine R. Lane, Leandro Nascimento Lemos, Lisa Karstens, Mark McCauley, Mitiku Mihiret Seyoum, Moamen M. Elmassry, Mustafa Guzel, Reid Longley, Simon Roux, Thomas M. Pitot, Emiley A. Eloe-Fadrosh

**Affiliations:** ^1^ Bioscience Division, Los Alamos National Laboratory, Los Alamos, NM, United States; ^2^ Department of Microbiology, Genetics, and Immunology, Michigan State University, East Lansing, MI, United States; ^3^ Department of Marine Sciences, University of Georgia, Athens, GA, United States; ^4^ In-Pipe Technology, Wood Dale, IL, United States; ^5^ Pacific Northwest National Laboratory, Richland, WA, United States; ^6^ Biological and Environmental Sciences and Engineering Division, King Abdullah University of Science and Technology (KAUST), Thuwal, Saudi Arabia; ^7^ Department of Earth and Environmental Sciences, University of Michigan, Ann Arbor, MI, United States; ^8^ Public Health Research Center, New York University Abu Dhabi, Abu Dhabi, United Arab Emirates; ^9^ Department of Biological Sciences, University of Calgary, Calgary, AB, Canada; ^10^ Department of Microbiology and Plant Pathology, University of California, Riverside, Riverside, CA, United States; ^11^ ICMR-CRMCH, National Institute of Immunohaematology, Chandrapur Unit, Chandrapur, Maharashtra, India; ^12^ Academy of Scientific and Innovative Research (AcSIR), Ghaziabad, Uttar Pradesh, India; ^13^ Department of Biology, Indiana University, Bloomington, IN, United States; ^14^ Department of Biology, Cell, and Molecular Biology, San Diego State University, San Diego, CA, United States; ^15^ DOE Great Lakes Bioenergy Research Center, San Diego State University, San Diego, CA, United States; ^16^ Department of Microbiology and Immunology, Wake Forest University School of Medicine, Winston-Salem, NC, United States; ^17^ Bioinformatics Laboratory, National Laboratory for Scientific Computing, Quitandinha, Rio de Janeiro, Brazil; ^18^ Scripps Institution of Oceanography, UC San Diego, La Jolla, CA, United States; ^19^ Graduate School of Genome Science and Technology, University of Tennessee, Knoxville, Knoxville, TN, United States; ^20^ Department of Microbiology and Plant Pathology, University of California: Riverside, Riverside, CA, United States; ^21^ Massachusetts Institute of Technology, Cambridge, MA, United States; ^22^ Ilum School of Science, Brazilian Center for Research in Energy and Materials (CNPEM), Campinas, São Paulo, Brazil; ^23^ Division of Oncological Sciences, Department of Obstetrics and Gynecology, Knight Cancer Institute, Oregon Health and Science University, Portland, OR, United States; ^24^ The Whitney Laboratory for Marine Bioscience and Sea Turtle Hospital, University of Florida, St. Augustine, FL, United States; ^25^ Department of Poultry Science, University of Arkansas, Fayetteville, AR, United States; ^26^ Department of Molecular Biology, Princeton University, Princeton, NJ, United States; ^27^ Department of Food Engineering, Hitit University, Corum, Türkiye; ^28^ Lawrence Berkeley National Laboratory, Berkeley, CA, United States; ^29^ Department of Biochemistry, Microbiology, and Bioinformatics, Université Laval, Québec, QC, Canada

**Keywords:** microbiome, multi-omics, data reuse, FAIR data, survey, metadata, data standards

## Abstract

Microbiome research is becoming a mature field with a wealth of data amassed from diverse ecosystems, yet the ability to fully leverage multi-omics data for reuse remains challenging. To provide a view into researchers’ behavior and attitudes towards data reuse, we surveyed over 700 microbiome researchers to evaluate data sharing and reuse challenges. We found that many researchers are impeded by difficulties with metadata records, challenges with processing and bioinformatics, and problems with data repository submissions. We also explored the cost constraints of data reuse at each step of the data reuse process to better understand “pain points” and to provide a more quantitative perspective from sixteen active researchers. The bioinformatics and data processing step was estimated to be the most time consuming, which aligns with some of the most frequently reported challenges from the community survey. From these two approaches, we present evidence-based recommendations for how to address data sharing and reuse challenges with concrete actions for future work.

## 1 Introduction

Researchers investigating microbiomes, whether from host, plant, water, or soil ecosystems, collectively generate large amounts of data increasingly from multi-omics experiments. The current paradigm in the field is to generate data for a given scientific question, yet the nature of multi-omics data often lends itself to reuse for data exploration and discovery purposes outside of the original study. More recent studies have demonstrated the tremendous value of a data reuse approach, including meta-analyses and comparative (meta)genomics, modeling efforts, and machine learning training (Duvallet et al., 2017; Kiledal et al., 2021; Madrigal et al., 2022; Saucedo et al., 2024; Li et al., 2025; Abdill et al., 2025; Elmassry et al., 2025). Microbiome data reuse has also enabled researchers to address their scientific questions at broader scales with data that they would not normally be able to generate themselves, such as continental and global-scale data (Zhang and Ning, 2015; McCauley et al., 2023; Lang et al., 2023; Osburn et al., 2024; Graham et al., 2024; Abdill et al., 2025), and difficult to acquire samples such as those from remote terrestrial and marine locations and even the International Space Station (Saunders and Rocap, 2016; Alexander et al., 2023; Pitot et al., 2024; Gonzalez et al., 2024; Nastasi et al., 2024). Reuse of published microbiome data has enabled the discovery of novel organisms and relationships, and informed our collective understanding of the biogeography of microorganisms and genetically encoded traits such as secondary metabolite production (Parks et al., 2017; Nayfach et al., 2021; Robinson et al., 2021; Edgar et al., 2022; Lima et al., 2022; Sanders et al., 2023; Machado et al., 2024; Elmassry et al., 2025).

To facilitate microbiome data reuse, several calls have been made to promote standardization, open data, and to increase data sharing (Gomez-Cabrero et al., 2014; Bhandary et al., 2018; Huttenhower et al., 2023; Sielemann et al., 2020; Eckert et al., 2020). More research teams, primary repositories, and institutions are promoting data reuse to facilitate enhanced analyses across samples, geographic locations, data types, and time scales (Jurburg et al., 2024). However, a more nuanced view into researchers’ behavior and attitudes towards data reuse, along with the associated costs, have not been explored in depth.

Here, we conducted a community survey of over 700 microbiome researchers to evaluate data sharing and reuse challenges. Based on the survey results, we next explored the cost and personnel time constraints of data reuse at each step of the data reuse process to better understand the “pain points” that could be improved upon. Together, we present recommendations for how to address these challenges and concrete actions to improve how the research community can further leverage data reuse for microbiome science.

## 2 Community analysis of barriers to microbiome data reuse

**FIGURE 1:**
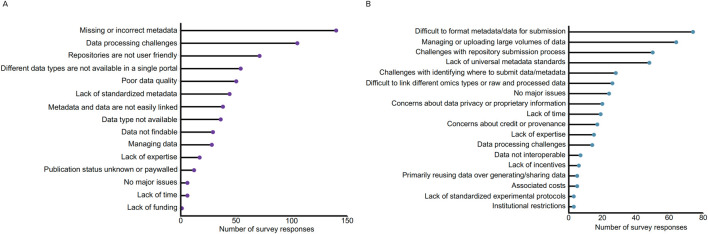
Synthesis of results from the community survey about challenges for data discovery and sharing. The answers to both free-form questions were classified into the broader categories for **(A)** data search: respondents were asked, “What is currently your biggest challenge when searching for microbiome data in available resources?” and **(B)** data sharing: respondents were asked, “What is currently your biggest challenge with respect to sharing microbiome data?”. All respondent answers and bins are available here: https://doi.org/10.5281/zenodo.14948343 (Kelliher et al., 2025).

To better understand the barriers to microbiome data reuse that researchers face, we conducted a survey in 2020 to assess various aspects of microbiome science. The survey was designed by the National Microbiome Data Collaborative (NMDC) team and reviewed and approved by the Human Subjects Committee at Lawrence Berkeley National Laboratory as an exempt IRB protocol under #394NR001. A total of 783 participants participated in the survey spanning 60 countries with approximately 50% of participants indicating they resided in the United States (415 out of 783 participants). Survey participants were asked a series of questions about their data sharing and data reuse practices, with the survey questions and anonymized results publicly available [https://doi.org/10.5281/zenodo.14948343] (Kelliher et al., 2025). Beyond the multiple choice questions, we specifically were interested in gathering feedback to understand the biggest challenges for (a) searching for microbiome data in available resources and (b) sharing microbiome data (Figure 1). For data search, we received responses from participants that outlined 637 challenges, with a plurality of responses (22%, 140/637) describing missing or incorrect metadata (Figure 1A). Other related issues with metadata were also reported, specifically a lack of standardized metadata (e.g., different ontologies and requirements across repositories) making it challenging to find data (7%, 44/637), along with issues linking primary data to the metadata (6%, 38/637). The next two categories with the most responses included challenges with processing data (16%, 105/637) and the user-friendliness of data repositories (11%, 71/637). Data processing challenges included issues with a lack of data interoperability (e.g., a lack of standardized formatting hindering data processing and different workflows leading to different outputs) and bioinformatics limitations (e.g., compute power, quality checks after data retrieval, downloaded data is not in a useful format for programmatic usage).

Survey feedback regarding “user-friendliness of data repositories” focused on issues with filtering or searching for data, a lack of interoperability between user interfaces and platforms, lack of programmatic access for downloading files, and issues with repositories or databases not being maintained. Related to challenges with data, many respondents specifically noted poor quality data (8%, 50/637) and difficulties managing data (4%, 28/637). Other response categories encompassed data accessibility, including the inability to find specific data of interest (Data type not available - 6%, 36/637; e.g., research area too niche, researchers not sharing data), concerns regarding data findability (5%, 29/637; e.g., challenges identifying relevant datasets, sorting through vast amounts of datasets, concerns about missing relevant datasets), or limited publication access (e.g., paywalls or datasets and publications not linked) to identify the study context (2%, 12/637). Lack of expertise was reported as one of the least limiting factors (3%, 17/637), while the lack of time or funding together only garnered 1% (n = 7) of 637 responses.

For data sharing, we received 428 separate issues. The top responses to this question related to issues submitting data, including difficulties in formatting metadata/data for submission (17%, 74/428), managing or uploading large volumes of data (15%, 64/428), and general challenges with repository submission processes (12%, 50/428) (Figure 1B). Related to difficulties formatting metadata, many responses specified that a lack of universal metadata standards (11%, 48/428) hindered the sharing process (e.g., uncertainty about which standards or ontologies to use). Other issues related to data management included difficulty linking raw and processed data or linking different omics types (6%, 26/428) (e.g., repositories not accepting different omics data types) and challenges navigating and choosing where to submit their data from the vast amount of repositories (7%, 28/428). Participants also reported concerns about data privacy (5%, 20/428) and concerns about credit or provenance (4%, 17/428), indicating that issues surrounding data reuse ethics may contribute to reduced data sharing. Lack of time (4%, 19/428), expertise (4%, 15/428), and incentives (1%, 6/428) were additional limiting factors, with researchers reporting that the data deposition process is time-consuming and tedious, and that there are insufficient resources for navigating proper data management and repository submissions. Similar to the responses regarding data reuse, the associated costs were not reported as a major issue (1%, 5/428).

Taken together, the responses to both data search and sharing indicate that challenges with metadata, data repositories, data management, and data processing represent major issues that limit effective data reuse across the microbiome research field.

## 3 A case study to assess the costs of reusing microbiome data

**FIGURE 2:**
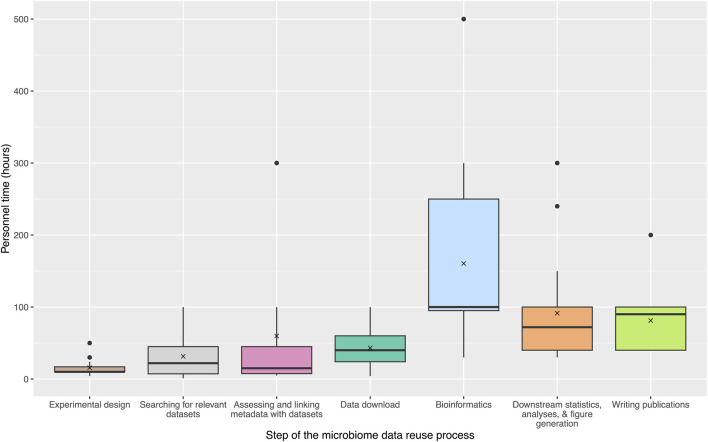
Estimates of personnel time for microbiome data reuse steps. These steps include: Experimental design, Searching for relevant datasets, Assessing and linking metadata with datasets, Data download, Bioinformatics, Downstream statistics, analyses, and figure generation, and Writing publications. Estimates were gathered from 16 NMDC Champions. Several Champions estimated “greater than” the hours that were used in the final average and median calculations (e.g., >100 h was treated as 100 h). Figure was generated using the ggplot2 R package and the *microshades* color palette (Wickham, 2011; Dahl et al., 2022).

To expand upon the community survey results and assess the costs associated with each step of the typical microbiome data reuse process, we collated information from active microbiome researchers part of the NMDC Champions program (https://microbiomedata.org/community/championsprogram/). Sixteen researchers provided estimates of personnel time and other resources associated with each step of the data reuse process from their own experiences. Figure 2 outlines the estimated personnel hours (excluding salaries or other associated costs) for each step. Personnel time was emphasized because it allowed for more direct comparisons between microbiome studies, regardless of institution or salary level, and could be used as a proxy for cost and burden estimations. Sixteen Champions assessed their “level of expertise” for large-scale data reuse (7 Intermediate and 9 Expert), and estimated the personnel time investment required at each research step, as well as the required computational resources. These estimates widely varied for each step, but overall the bioinformatics step was reported as the largest time burden (average: 160.5 hours (h); median: 100 h), followed by the downstream statistics, analyses, and figure generation step (average: 91.5 h; median: 72 h) and the publication writing step (average: 81.25 h; median: 90 h). In the community survey, many researchers reported difficulties in managing and uploading large amounts of data. To further quantify the amounts of data involved in typical reuse studies, Champions estimated the amount of computational resources required for data storage as well as the computational resources required for data analysis and bioinformatics. A range from 1 TB to 10 TB was estimated for data storage, and up to 10,000 core hours were reported for data analysis and bioinformatics, although this metric was not able to be estimated by all Champions. Together, this case study to estimate time constraints and costs illustrates practical data reuse steps in a more quantitative way. Based on these data, we offer evidence-based recommendations to improve the process with an eye towards streamlining future data reuse.

## 4 Discussion

This view into researchers’ attitudes towards data reuse and cost estimates is instructive and allows for an enhanced understanding of how microbiome researchers can leverage existing data investments. By clarifying how challenges are perceived and the associated costs of data reuse, we are able to establish evidence-based recommendations for future work. Using a community survey approach, we found that there are several issues that disincentivize researchers from reusing data. One major theme of reported challenges in both sharing and reusing data involved metadata quality, standardization, and availability. This echoes other reports surrounding lagging adoption of metadata standards and best practices (Vangay et al., 2021; Cernava et al., 2022; Fraga-Gonzalez et al., 2025), further emphasizing that this is a barrier that needs to be universally addressed. Challenges with processing large volumes of data was reported in the community survey, and this burden was also reflected by the NMDC Champion estimates that the bioinformatics steps are the most time consuming and require large amounts of computational resources. Difficulties with repositories for data sharing as well as for finding and accessing reusable microbiome data were also reported.

The case study estimation analysis, while limited to sixteen individuals, provides more quantitative information on data reuse that, to our knowledge, has not been reported in other studies discussing barriers to data reuse. This analysis is meant as a preliminary assessment of current practices to elaborate upon discussion points that have been reported in other perspectives (Tenopir et al., 2011; Tenopir et al., 2015; Huttenhower et al., 2023). We recognize that other costs such as those associated with computational resources widely vary across institutions and that it can be difficult or impossible to obtain quotes or financial information to quantify and standardize cost assessments across the entire microbiome research community. We assessed personnel time requirements as a more comparable metric across microbiome research questions, researchers, and institutions, however this also has its limitations when used as a measure of burden.

### 4.1 Recommendations

Moving forward, it is increasingly clear that data reuse challenges must be addressed from the perspectives of both depositors and reusers to make it more broadly feasible. Below, we provide specific recommendations based on our synthesis of both the community survey and Champions feedback on data sharing, reuse, and costs.

### 4.2 Metadata

From our survey, researchers reported issues with metadata collection, standardization, reporting, deposition, and access as significant barriers to both sharing and reuse. The Genomic Standards Consortium (GSC), the Environment Ontology (EnvO), and the Open Biomedical Ontologies (OBO) Foundry are all examples of community-driven efforts that have significantly advanced how microbiome metadata can be collected and standardized (Field et al., 2011; Buttigieg et al., 2013; Smith et al., 2007). More recently, two large community-driven efforts have emerged to assist researchers with consistently reporting and publishing on microbiome data: the STORMS and STREAMS guidelines for human microbiome and environmental microbiome data, respectively (Mirzayi et al., 2021; Kelliher et al., 2024a). Despite these efforts, there is generally a lack of awareness of existing metadata standards and an even more pronounced lack of adoption (Vangay et al., 2021; Cernava et al., 2022). We suspect that this lack of awareness and utilization of metadata standards significantly contributes to the challenges the community faces with sharing and reusing data. Additional training, tutorials, and awareness of metadata and data standards would provide significant benefits at the individual and community levels to increase adoption and implementation of these efforts.

### 4.3 Finding and accessing data through data repositories

Searching for, finding, and accessing relevant datasets was emphasized as a pain point for researchers in both the community survey and as a time burden in the Champions’ estimates. Enhanced interoperability between datasets and repositories can assist researchers in these steps, and it is often the responsibility of the data submitter to ensure that there are decipherable connections between the data and metadata. Survey participants reported issues navigating repositories for both sharing and searching for data, and a lack of training or tutorials hindering this process. Educational resources with a focus on data repositories could enhance researchers’ ability to effectively share their data and adhere to FAIR [Findable, Accessible, Interoperable, and Reusable] principles, thus making data more accessible overall (Wilkinson et al., 2016). Several data repositories offer links to other related repositories or datasets which can help in the search process (Gebre et al., 2025). It can also be important to note whether repositories have been curated and in what manner to minimize the reported issues with insufficient data and metadata quality (Eloe-Fadrosh et al., 2022; Muller et al., 2022). Additionally, when publishing on data reuse studies, it is important to note that many repositories accept processed data, which can improve reproducibility of the published work (Baker et al., 2000; McWilliam et al., 2013). Researching and adhering to the data use policies and citing data from repositories properly can save time during revisions and can foster trust and incentives for those that report hesitancy with data sharing. Lastly, we anticipate newer tools like incorporating machine learning or artificial intelligence within repositories will also help to address challenges in data quality control for both raw and processed data (reviewed in Hernández Medina et al., 2022; Kumar et al., 2024).

### 4.4 Data processing and bioinformatics

Bioinformatics was reported as the most time-consuming step for the NMDC Champions. Data processing steps would be significantly less time consuming for researchers if free, publicly available software and tools were more readily available. Several web-based cyberinfrastructures exist that increase the accessibility of bioinformatics workflows (Swetnam et al., 2024; Lo et al., 2022; Arkin et al., 2018; Li et al., 2017; Kelliher et al., 2024b). For data download, tools such as the Sequence Read Archive (SRA) toolkit and Globus can help researchers to improve their download procedures (Foster and Kesselman, 1997; Chard et al., 2016; Heldenbrand et al., 2017; Sayers et al., 2022). More transfer services at the institutional and individual levels (such as the services provided by IMG/M) as well as more publicly available application programming interfaces (APIs) would also minimize data download burdens for researchers (Chen et al., 2023). Additional training, webinars, workshops, tutorials, and documentation would all lower the barriers to these steps, especially for researchers that are not as familiar with these processes. Publishing and sharing code used for data reuse can also facilitate collective improvements across the field.

## 5 Conclusion

Taken together, many of the responsibilities for promoting microbiome data reuse have been discussed from the perspective of the data generators (Huttenhower et al., 2023). Other new tools, resources, and recommendations can also improve the data reuse process and decrease researcher burden. Increased collaboration and discussions between data generators and reusers can also lead to the sharing and adoption of data and metadata best practices. We encourage the continuation of calls to action for increased reuse of microbiome data, ideally from the perspective of all organizational partners including research teams, data repository representatives, funding agencies, and publishers. Grant funding calls for research projects specifically reusing data and providing compute time may incentive more meta-analyses. While community awareness and adoption of FAIR and open data management practices is increasing, addressing the reported challenges will facilitate further implementation across the field. The two lines of investigation reported herein provide insight into the behaviors and practices of microbiome researchers, and the barriers they encounter with microbiome data reuse. This perspective contributes to our collective understanding of researcher attitudes towards data reuse, and provides recommendations for how the community can work together to address the most pressing challenges in microbiome data reuse.

## Data Availability

The datasets presented in this study can be found in online repositories. The names of the repository/repositories and accession number(s) can be found below: The data analyzed for this study can be found in Zenodo [https://doi.org/10.5281/zenodo.14948343].
